# Genome-Wide Identification and Integrative Analysis of the *Fruit-Weight 2.2-Like* Family Suggests Potential Roles in Fiber Development and Stress Responses in *Gossypium hirsutum*

**DOI:** 10.3390/biology15151282

**Published:** 2026-08-04

**Authors:** Jiaxin Zhang, Glory Enujioke, Xin Ruan, Yi Yu, Wenhui Song, Jin Peng, Fangjuan Chen, Zhengsheng Zhang, Xueying Liu

**Affiliations:** 1 College of Agronomy and Biotechnology, Southwest University, Chongqing 400715, China; 2 Engineering Research Center of South Upland Agriculture, Ministry of Education, Academy of Agricultural Sciences, Southwest University, Chongqing 400715, China

**Keywords:** *fruit-weight 2.2*, genome-wide identification, cotton, stress resistance, fiber-related traits, virus-induced gene silencing

## Abstract

The *Fruit-Weight 2.2-Like* (*FWL*) gene family plays crucial roles in determining organ size and stress responsiveness in plants. However, the *FWL* family members remain largely unexplored in *Gossypium* species. In this study, we identified and characterized the *FWL* family in *Gossypium hirsutum* to investigate their gene expansion, functional evolution, and potential association with key agronomic traits. A total of 51 *GhFWLs* were identified, and their gene structures, domain compositions, and phylogenetic relationships were comprehensively analyzed. Expression profiling under heat, cold, salt, and drought stress conditions revealed that most *GhFWLs* participate in abiotic stress response pathways. Haplotype-based association analysis revealed significant associations for 14 *GhFWLs* with fiber-related traits, suggesting their potential functions in regulating fiber development. Furthermore, silencing the family member *GhMCA1* provided supporting evidence for its role in salt stress tolerance. Our findings provided insightful information about the *FWL* gene family in *G. hirsutum* and highlighted candidate genes for improving fiber-related traits and stress tolerance through molecular breeding approaches.

## Simple Summary

The *Fruit-Weight 2.2-Like* gene family was systematically identified and characterized in cotton species for the first time. Our findings suggested the functional diversity of family members in their reactions to abiotic stress and the potential regulation of fiber-related traits, providing important candidate gene resources for further genetic improvement of cotton.

## 1. Introduction

Cotton is a vital cash crop in the world. Fibers, the main product of cotton, are the primary natural raw material of the textile industry. Additionally, the cottonseed meal and oil, as well as gossypol, are widely used in food, feed, and pharmaceutical fields [1,2]. Therefore, improving the yield and fiber quality of cotton has always been one of the primary objectives of cotton breeding programs worldwide. However, the reduction in arable land area and the escalating intensity of abiotic stressors, including drought, extreme temperatures, and soil saline-alkalization, cause significant hindrance to cotton production in the context of global climate change and population growth [3]. Given this circumstance, it is necessary to explore key genes or gene families that regulate fiber quality, yield, and stress resistance traits of cotton. Illustrating the functional mechanisms of these families/genes will offer useful resources for the genetic improvement of cotton and lay a foundation for ensuring the steady production and supply of the global cotton industry.

The *Fruit-Weight 2.2-Like* (*FWL*) gene family, also known as the *Cell Number Regulator* (*CNR*) gene family, is extremely conserved across eukaryotes. The representative domain of the FWL protein family is the PLAC8 (Placenta-specific 8), which contains one or two transmembrane regions as well as two cysteine/proline-rich regions [4]. The *FWL* gene family member in plants was initially identified as the candidate gene for a major QTL (Quantitative Trait Locus), *fruit-weight 2.2* (*fw2.2*), which accounts for approximately 30% of the variation in fruit weight in tomato [5,6]. According to molecular mechanism studies, *FW2.2* is involved in the spatiotemporal regulation of callose deposition at plasmodesmata and negatively regulates cell division [7,8]. Its conserved function in regulating plant growth and development has been revealed by further research on the *FWL* gene family in a variety of plants. For instance, *PfCNR1* was negatively correlated with cell division in the ovaries of *Physalis floridana* [9]. In maize, *ZmCNR1* and *ZmCNR13* have both been reported to affect plant height and ear size [10,11]. *OsCNR1* determined grain width and weight in rice [12], and *OsFWL4* could negatively regulate the tiller number and plant yield [13].

In addition to regulating plant development, several *FWL*/*CNR* family genes have been shown to be involved in ion homeostasis and abiotic stress response [14]. A special clade of the *FWL* gene family comprises genes that function as mechanosensitive channels, mediating stress response by influencing Ca^2+^ influx. For instance, *AtMCA1* and *AtMCA2* have been reported to contribute to the transient elevation of cytosolic Ca^2+^ upon cold shock [15]. *OsMCA1*, together with *NtMCA1* and *NtMCA2*, participates in the regulation of plasma membrane Ca^2+^ uptake and ROS (Reactive Oxygen Species) accumulation under hypoosmotic stress [16,17]. Other members of the *FWL* gene family are more likely to participate in the regulation of other metal ions. For example, *AtPCR2* was crucial for zinc (Zn) redistribution and Zn detoxification [18]. By repressing Cd^2+^ uptake and accumulation, *SICNR8* enhanced cadmium (Cd) resistance in transgenic poplar [19]. Overexpression of *TuCNR10* in *Arabidopsis* improved Cd, Zn, and manganese (Mn) tolerance [20].

Although the functions of *FWL* family genes have been characterized in various plants [14,21,22], related studies remain limited in cotton, and its potential functions in fiber development and stress responses remain largely unexplored. *Gossypium hirsutum*, an allotetraploid containing the At subgenome and Dt subgenome, is the most widely cultivated cotton species. Recent genome-wide family studies have illustrated that integrating evolutionary, transcriptomic, and association data enables efficient prioritization of candidate genes for trait improvement [23,24,25]. Given the known roles of their homologs in other plants, we hypothesized that certain FWL members may act as regulators of stress response and fiber development. To address this, *FWL* gene family members were genome-wide identified in *Gossypium hirsutum* for the first time in this study. A comprehensive analysis of their chromosomal distribution, structural features, phylogenetic relationships, expression profiles, and associations with fiber-related traits was conducted. Findings in this study provided valuable insights into the potential role of *GhFWLs* and laid a foundation for genetic improvement in cotton.

## 2. Materials and Methods

### 2.1. Identification of FWL Genes in G. hirsutum

The protein sequences of *G. hirsutum* (TM-1_V2.1) were obtained from the COTTONOMICS database (http://cotton.zju.edu.cn/, accessed on 1 June 2026). The protein sequences of *G. herbaceum* (A1_WHU) and *G. raimondii* (D5_HAU) were obtained from the CottonMD database (https://yanglab.hzau.edu.cn/CottonMD.1, accessed on 1 June 2026) [26]. The Hidden Markov Model (HMM) profile of the PLAC8 domain (PF04749) was downloaded from the Pfam website (http://pfam.xfam.org/, accessed on 8 June 2026) [27]. The ‘hmmersearch’ module in HMMER 3.0 [28] software was used to screen the TM-1 proteome with an e-value threshold of 1.0 × 10^−5^, following the official user guide. To validate the presence of the PLAC8 domain, the obtained protein sequences of FWL family candidates were then submitted to the Conserved Domain Database (CDD, https://www.ncbi.nlm.nih.gov/cdd, accessed on 8 June 2026). Proteins, identified by HMMER 3.0 and confirmed by CDD, were determined as members of the FWL protein family. Chromosomal locations of *GhFWLs* were retrieved from reference genome annotation files and visualized using the R package “RIdeogram” (v0.2.2) [29].

### 2.2. Molecular Characterization of FWL Genes/Proteins in G. hirsutum

Gene lengths and structural information of *GhFWL* genes were also retrieved from the TM-1_V2.1 reference genome annotation files deposited in the COTTONOMICS database. The ExPASy ProtParam tool (https://web.expasy.org/protparam/, accessed on 10 June 2026) was used to analyze the physicochemical properties of GhFWL proteins. The conserved motifs and subcellular locations of GhFWL proteins were predicted by the MEME suite (https://meme-suite.org, accessed on 10 June 2026) [30] and the Plant-PLoc server (http://www.csbio.sjtu.edu.cn/bioinf/plant-multi/, accessed on 10 June 2026) [31], respectively. MEME was executed with the maximum number of motifs set to 10, motif width ranging from 6 to 50 amino acids (aa), and the default e-value threshold applied [30]. For the identification of cis-acting elements, 2000 bp upstream promoter sequences of *GhFWLs* were analyzed using the PlantCARE database (https://bioinformatics.psb.ugent.be/webtools/plantcare/html/, accessed on 15 June 2026) [32]. Figures about gene structures, conserved motifs, and domains were created using TBtools (v2.450) [33], while the R package “ComplexHeatmap” (v2.29.0) [34] was used to generate the heatmap of cis-acting elements.

### 2.3. Phylogenetic and Collinearity Analysis and Calculation of Ka/Ks Values of FWL Genes in Cotton and Three Other Dicot Species

FWL protein family members from three dicot species (*Glycine max*, *Solanum lycopersicum*, and *Arabidopsis thaliana*) were combined with *G. hirsutum* FWL proteins for multiple sequence alignment using ClustalW implemented in MEGA 11 software. Protein sequences of *G. max*, *S. lycopersicum*, and *A. thaliana* were obtained from the study of Ran et al. (2023) [22]. An unrooted phylogenetic tree was constructed using the Neighbor-Joining method in MEGA 11 with 1000 bootstrap replicates. The online tool iTOL (https://itol.embl.de/, accessed on 15 June 2026) was used to visualize and optimize the tree [35]. The “One-Step MCScanX” and “Simple Ka/Ks calculator” algorithms integrated with TBtools [33] were utilized to analyze the collinearity between *FWL* genes and to calculate their Ka/Ks values, respectively. Pairs with Ks = 0 were excluded from the analysis.

### 2.4. Expression Analysis of GhFWLs in Different Tissues and Under Abiotic Stress

The expression patterns of *GhFWL* genes across various tissues were obtained from the COTTONOMICS database based on the investigation of Hu et al. (2019) [36]. Besides developing ovules and fibers, tissues including roots, stems, leaves, petals, anthers, and pistils were also included for expression analysis. In addition, transcript profiles from leaves under heat, cold, salt, and drought stress (simulated by polyethylene glycol, PEG) were analyzed to explore the potential roles of *GhFWLs* in regulating stress response. The R package “ComplexHeatmap” was used to visualize their expression patterns and changes using the values of log_2_(FPKM+1) and log_2_(fold change), respectively.

### 2.5. Association Analysis of GhFWLs with Fiber-Related Traits

The genetic variation data of *GhFWLs* and phenotypic information of six fiber-related traits retrieved from the CottonMD database (https://yanglab.hzau.edu.cn/CottonMD.1, accessed on 1 June 2026) [26] were used for association analysis. Among these datasets, phenotypic data for four fiber quality traits (fiber length, strength, micronaire, and elongation) were derived from He et al. (2021) [37], a study that included 1285 cotton accessions. Meanwhile, data for the other two yield-related traits, including lint percentage and lint weight per boll, were obtained from Guo et al. (2021) [38], a study that included 503 accessions. The statistical method and parameters for association analyses were based on the default parameters of the CottonMD database (https://yanglab.hzau.edu.cn/CottonMD.1, accessed on 1 June 2026) [26].

### 2.6. Plant Materials and Growing Conditions

*G. hirsutum* cultivar YZ-1 was used for the virus-induced gene silencing (VIGS) assay in this study. Plants were grown in Hoagland’s nutrient solution in a growth chamber (Ningbo Le Electrical Instrument Manufacturing Co., Ltd., Ningbo, China) under controlled conditions: a temperature of 25 °C, a 16 h light/8 h dark photoperiod, 70% relative humidity, and a light intensity of 22,000 lx. For salt stress treatment, cotton seedlings at the four-leaf stage were treated with a 200 mM NaCl solution for 12 h.

### 2.7. Virus-Induced Gene Silencing Assay

A specific fragment targeting the *FWL* family member *GH_A02G1993* was amplified and cloned into the pTRV2 vector. A 250 bp fragment for VIGS was selected to minimize off-target effects. Primer sequences for vector construction were listed in Table S5. The *CLA1* gene was used as a positive control for silencing efficiency, and the empty vector TRV2:00 served as the negative control [39]. The recombinant vectors were transformed into *Agrobacterium tumefaciens* strain GV3101 produced by Weidi Biology Co., Ltd. (Shanghai, China). *Agrobacterium* cultures were grown to OD_600_ = 1.0. Subsequently, TRV1- and TRV2-containing cultures were mixed at a 1:1 ratio and infiltrated into the cotyledons of YZ-1 seedlings. The infiltrated plants were maintained in darkness at 22 °C for 48 h and subsequently returned to the original growth chamber conditions. Silencing efficiency was assessed by RT-qPCR at 14 days after infiltration. Moreover, 0.1% 3,3′-Diaminobenzidine (DAB) staining was applied to detect peroxidase accumulation under salt stress [40].

### 2.8. RNA Extraction and RT-qPCR Assay

The total RNA was extracted from leaves of the infiltrated plants using the StarSpin Plant RNA Kit (Polysaccharides and Polyphenols-rich reagent; GenStar, Beijing, China). First-strand cDNA was synthesized using the StarScript II RT Kit (GenStar, Beijing, China). Briefly, 1 µg of total RNA was mixed with 1 µL of StarScript II Enzyme Mix and 10 µL of 2 × StarScript II Buffer, and the mixture was made up to a final volume of 20 µL with nuclease-free water. The reaction mixture was incubated at 42 °C for 15 min, followed by heat inactivation at 85 °C for 5 min to generate cDNA. Quantitative reverse transcription PCR (RT-qPCR) was then performed using the cDNA as a template with the 2 × RealStar Fast SYBR qPCR Mix (GenStar, Beijing, China). In detail, 2 µL diluted cDNA was combined with 10 µL 2 × RealStar Fast SYBR qPCR Mix, 0.5 µL primer pair for each, and 7 µL sterile water. The two-step qPCR program was set as follows: initial denaturation at 95 °C for 5 min, followed by 40 cycles consisting of denaturation at 95 °C for 10 s and combined annealing/extension at 60 °C for 30 s. The relative expression levels of target genes were calculated using the 2^−Δct^ method [41], with *GhActin* serving as the reference gene. Each treatment included five biological replicates and three technical replicates. The sequences of primers used were listed in Table S5.

## 3. Results

### 3.1. Genome-Wide Identification and Characterization of the FWL Gene Family in Cotton

**Figure 1:**
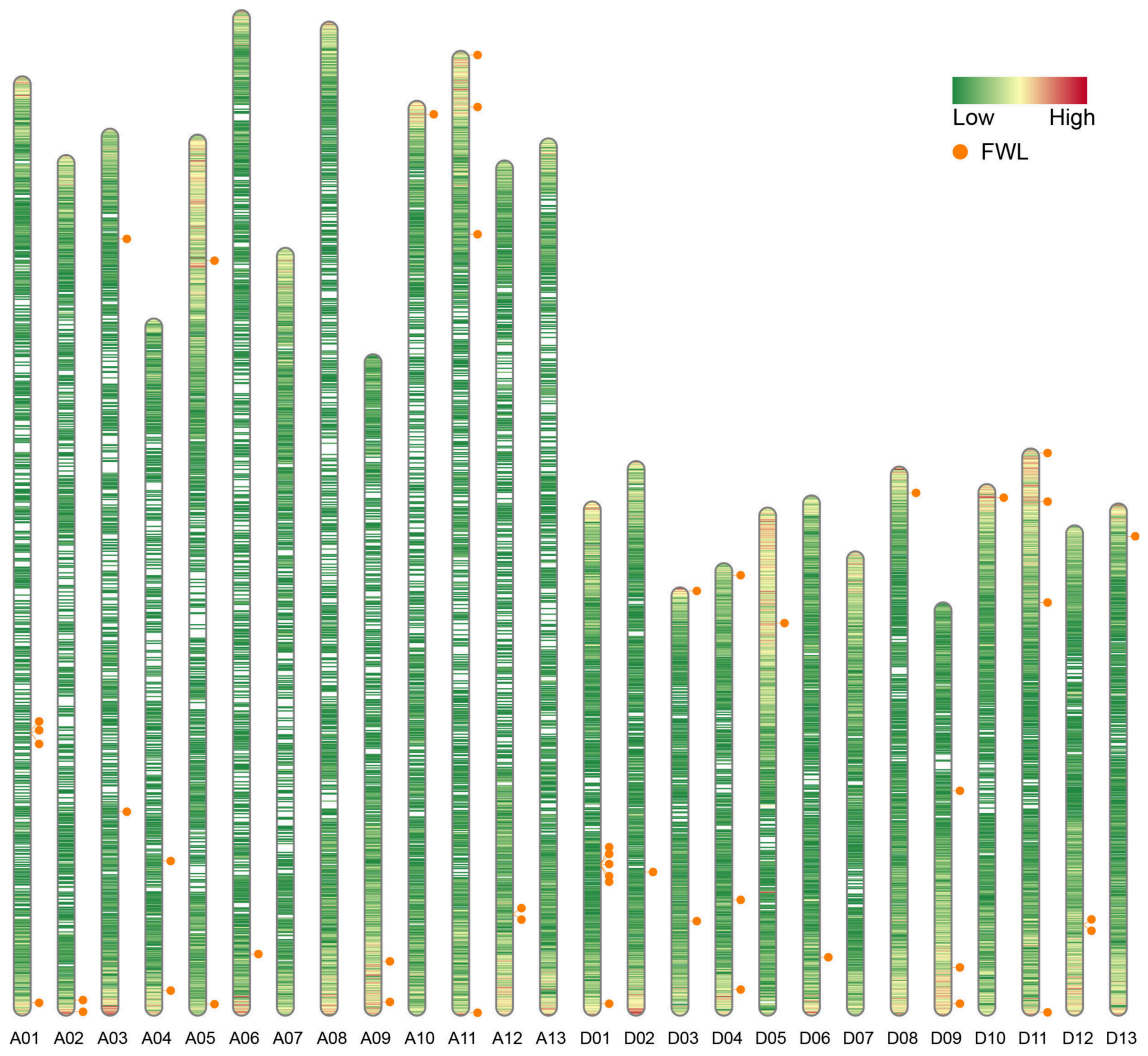
Chromosomal distribution of *GhFWL* gene family members in *G. hirsutum.* (Chromosomes were ordered by their respective chromosome numbers within each subgenome. Gene density across chromosomes was represented by a color gradient from green (low density) to red (high density). Orange dots marked the physical positions of *GhFWL* genes. Three *GhFWL* genes located on an unassembled scaffold of Chromosome D05 were not included in this figure).

A total of 51 *FWL* gene family members were preliminarily identified in *G. hirsutum* based on the HMM profile of the conserved PLAC8 domain (PF04749). All candidate genes were further validated via NCBI-CDD, confirming the presence of the PLAC8 domain in all identified sequences. Chromosomal distribution analysis revealed uneven localization of *GhFWL* genes across 22 chromosomes, with 22 members encoded by the At subgenome and 29 by the Dt subgenome (Figure 1). Chromosome D01 harbored the maximum number of *GhFWL* genes (six members), followed by chromosomes A01, A11, D05, and D11, each containing four *GhFWL* genes. In contrast, no *GhFWL* genes were identified on chromosomes A08, A13, and D07.

The number of exons in *GhFWL* genes ranged from 1 to 8, and their genomic lengths varied from 436 to 6515 bp. Most GhFWL proteins contained 150 to 300 amino acids, while only ten members harbored more than 300 amino acids. The molecular weights of these proteins ranged from 8.45 to 65.5 kDa. Subcellular localization predictions indicated that GhFWL proteins were predominantly localized to the cell membrane and vacuole, whereas five members were distributed in the cytoplasm. This subcellular distribution pattern suggested functional diversity of the GhFWL protein family in *G. hirsutum*. Detailed basic physicochemical features of all *GhFWL* members were summarized in Table S1.

To understand the numerical disparity between the At and Dt subgenomes, we performed a genome-wide identification of the *FWL* gene family in two diploid species, *G. herbaceum* and *G. raimondii*, which are considered the progenitors of *G. hirsutum*. As a result, 29 and 28 *FWL* genes were identified in *G. herbaceum* and *G. raimondii*, respectively (Figure S1, Tables S2 and S3). This approximately equal number suggested that gene loss events within the *FWL* gene family might have occurred in the At subgenome following the polyploidization of *G. hirsutum*. Furthermore, we calculated the Ka/Ks value of homologous *FWL* gene pairs between the following: the At subgenome and *G. herbaceum*; the Dt subgenome and *G. raimondii*; and the At and Dt subgenomes of *G. hirsutum*. After excluding pairs with Ks = 0 (10 pairs), the remaining *FWL* homologous pairs were analyzed for Ka/Ks ratios. The distribution of Ks values ranged from 0.004 to 3.634. As shown in Table S4, the vast majority (89.6%) of these pairs exhibited Ka/Ks values below 0.5, suggesting that they underwent purifying selection during evolution. Of the remaining *FWL* gene pairs, three showed evidence of positive selection (Ka/Ks > 1), while seven appeared to be under relaxed purifying selection (0.5 < Ka/Ks < 1).

### 3.2. Phylogenetic and Synteny Analysis of the FWL Family Among Four Dicotyledonous Species

**Figure 2:**
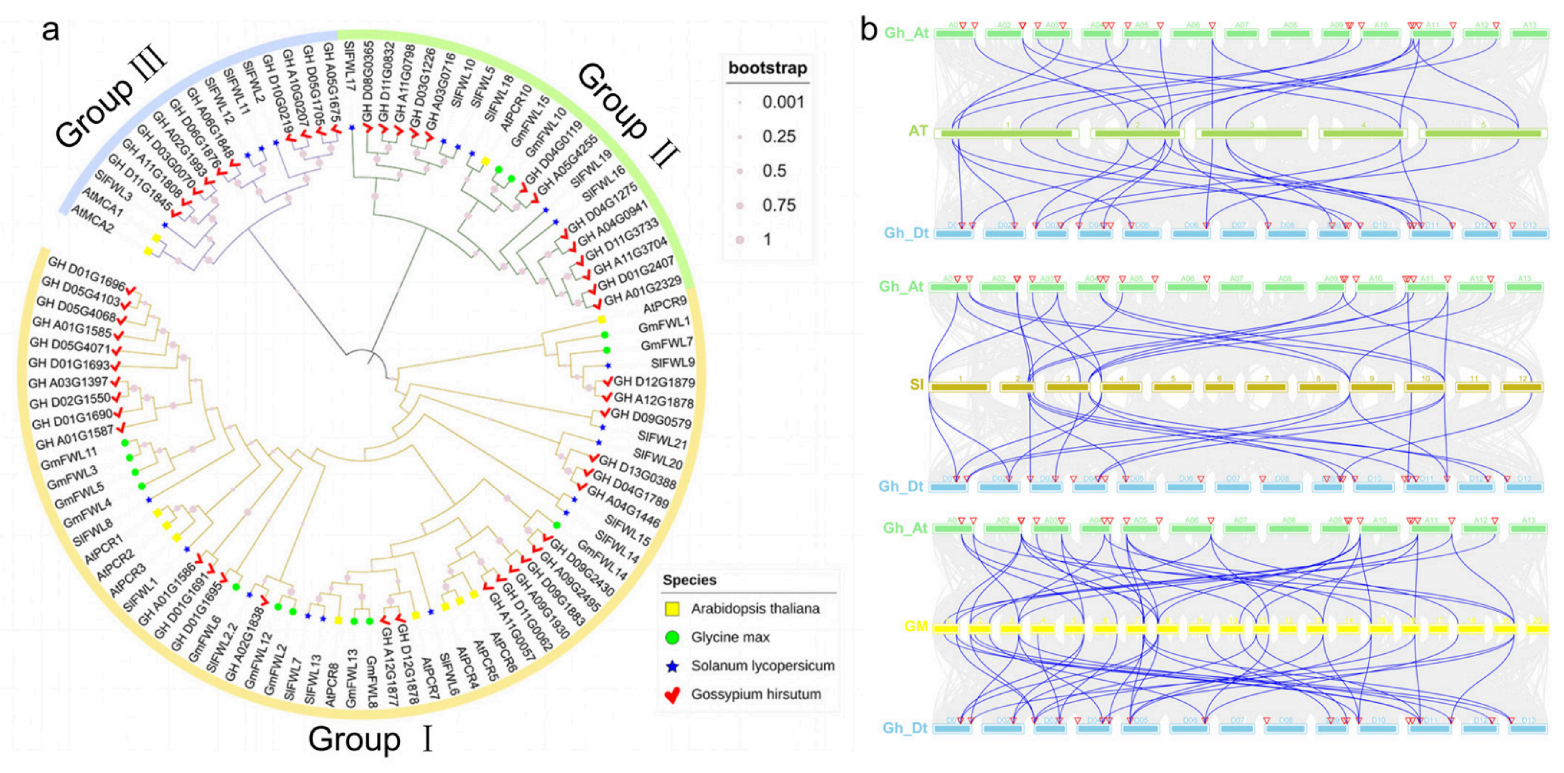
Phylogenetic and synteny analysis of the FWL protein family. (**a**) Phylogenetic analysis of the FWL protein family from *G. hirsutum*, *Arabidopsis thaliana* (At), *Glycine max* (Gm), and *Solanum lycopersicum* (Sl). Protein sequences of *A. thaliana*, *G. max*, and *S. lycopersicum* were obtained from Ran et al. (2023) [22]. (**b**) Collinearity analysis of *GhFWL* genes with the other three species. Numbers indicate bootstrap values, as explained in figure. Triangles represent FWL proteins, with gray lines in the background represented the overall genome synteny, while blue lines highlighted syntenic gene pairs.

Previous studies have demonstrated distinct evolutionary patterns of the *FWL* gene family between monocot and dicot plants [14]. To investigate the evolutionary relationships and functional conservation of *FWL* family members in upland cotton, a phylogenetic tree was constructed based on FWL protein sequences from upland cotton and three other dicot species, including tomato (*Solanum lycopersicum*), soybean (*Glycine max*), and *Arabidopsis* (*Arabidopsis thaliana*) (Figure 2a). In total, 98 FWL proteins were included in the phylogenetic tree, consisting of 51 from upland cotton, 21 from tomato, 14 from soybean, and 12 from *Arabidopsis*. Phylogenetic analysis clustered these FWL proteins into three distinct groups. Group I represented the largest clade, containing 60 FWL proteins, among which 28 were GhFWL protein members. Group II contained 22 proteins, with a species distribution of one from *Arabidopsis*, two from soybean, six from tomato, and 13 from cotton. Group III comprised 16 members derived from Arabidopsis, tomato, and cotton, but not soybean, with 10 members originating from upland cotton. Collectively, these findings revealed that GhFWL proteins shared conserved evolutionary features with other dicots while undergoing unique lineage-specific expansions during cotton evolution.

To further elucidate the evolutionary mechanisms underlying the expansion of the *FWL* family in cotton, we analyzed the syntenic relationships of *FWL* genes between upland cotton and the other three dicot species (Figure 2b). A total of 153 orthologous pairs were identified. The highest number of pairs was observed between cotton and soybean (72 pairs, accounting for 47.1% of the total), followed by cotton with tomato (41 pairs, 26.8%) and cotton with *Arabidopsis* (40 pairs, 26.1%). Among the 51 *GhFWL* members, 42 (82.4%) possessed syntenic orthologs with at least one other species, indicating a high degree of evolutionary conservation within dicots. Notably, the remaining 9 *GhFWL* members (*GH_A01G1586*, *GH_A01G1587*, *GH_D01G1691*, *GH_D01G1693*, *GH_D01G1695*, *GH_D01G1696*, *GH_D05G4068*, *GH_D05G4071*, and *GH_D05G4103*) lacked orthologous pairs in the other three species. These genes were tandemly distributed on chromosomes A01, D01, and D05 and exclusively clustered in Group I, suggesting a lineage-specific expansion via tandem duplication. Furthermore, 19 *GhFWL* members, including three from Group I, eight from Group II, and eight from Group III, exhibited synteny with all three dicot species. These genes likely represent the highly conserved core members of the *FWL* family, which may undertake essential and fundamental biological functions in dicot plants.

Collectively, our phylogenetic and syntenic findings elucidated that widespread segmental duplications and tandem duplications jointly have contributed to the overall expansion of *FWL* gene family members in cotton.

### 3.3. Conserved Motifs, Domains, and Gene Structures Analysis of GhFWLs

**Figure 3:**
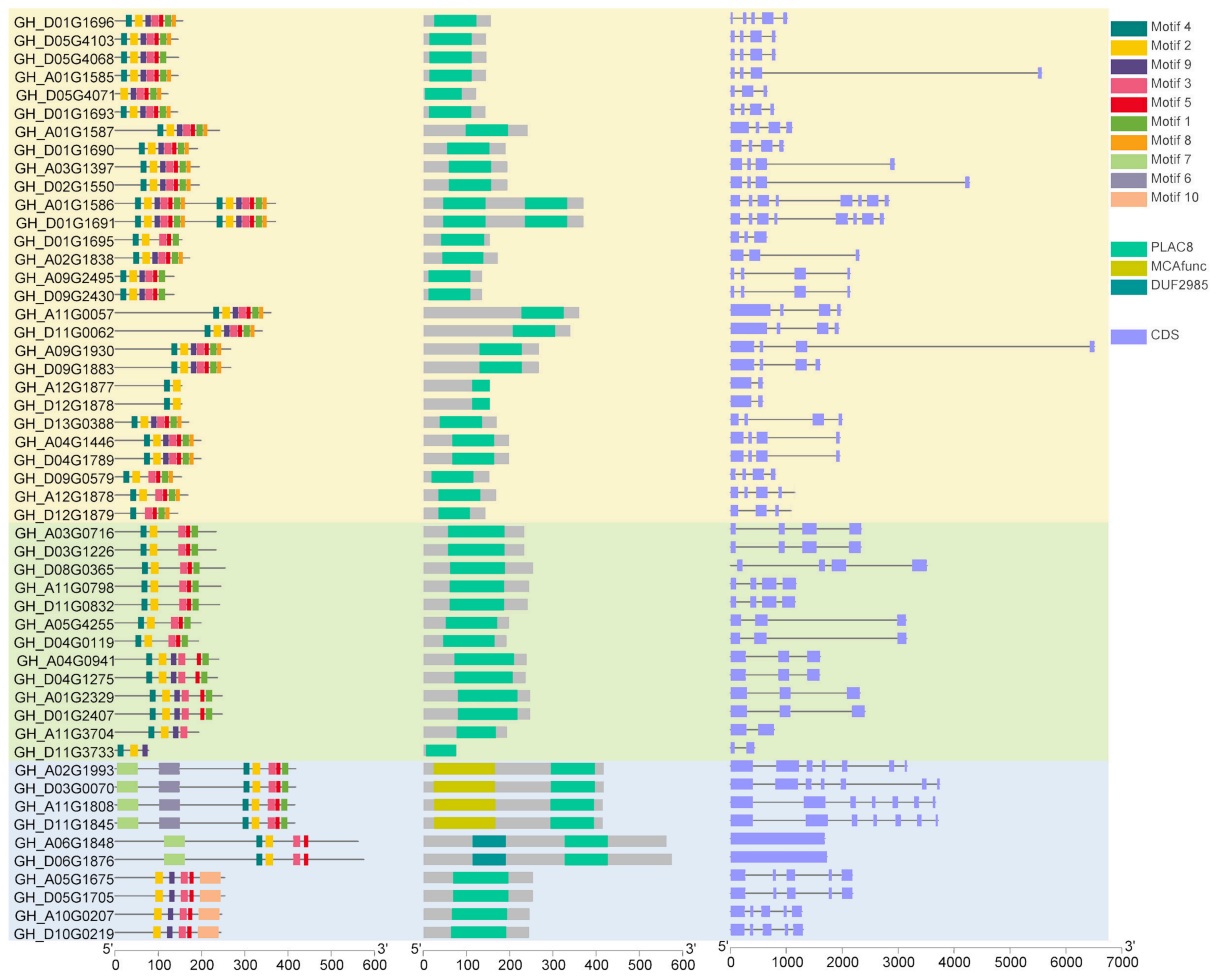
Conserved protein motifs (**left**), functional domains (**middle**), and gene structures (**right**) of GhFWL proteins.

Gene structures, conserved motifs, and domains are core characteristics for illustrating the evolutionary patterns and functional diversity of gene families. Analysis of the gene structures of *GhFWLs* revealed that 76.5% of the members contain 3–5 exons (Figure 3). Among the remaining members, four *GhFWLs* containing seven exons and two intronless *GhFWLs* belonged to Group III, while two *GhFWLs* with eight exons were derived from Group I.

Motif composition analysis (10 motifs in total, Figure S2) indicated that six motifs (Motifs 1–5 and 9) were the core motifs of GhFWL proteins, as they were present in all three groups. In contrast, Motifs 6, 7, and 10 were exclusively identified in Group III members, and Motif 8 was unique to Group I, implying clade-specific functional innovations (Figure 3). Furthermore, domain analysis demonstrated that the members from Group I and Group II contained only the PLAC8 domain, while eight members in Group III possessed an additional domain (MCAfuc or DUF2965) alongside PLAC8 (Figure 3). Notably, two homologous GhFWL protein pairs in Group I (*GH_A01G1586* and *GH_D01G1691*) contained two PLAC8 domains, in contrast to all other family members, which contained only one. Given their unique exon number (eight), distinctive motif arrangement (a tandemly duplicated arrangement of 7 motifs), and duplicated domain, we speculated that tandem duplication events occurred in these two genes prior to cotton tetraploidization. Overall, the strong correlation between phylogenetic grouping and gene structure or motif/domain composition highlighted both functional conservation and diversification among *GhFWL* genes.

### 3.4. Identification of Cis-Acting Elements in GhFWL Genes

**Figure 4:**
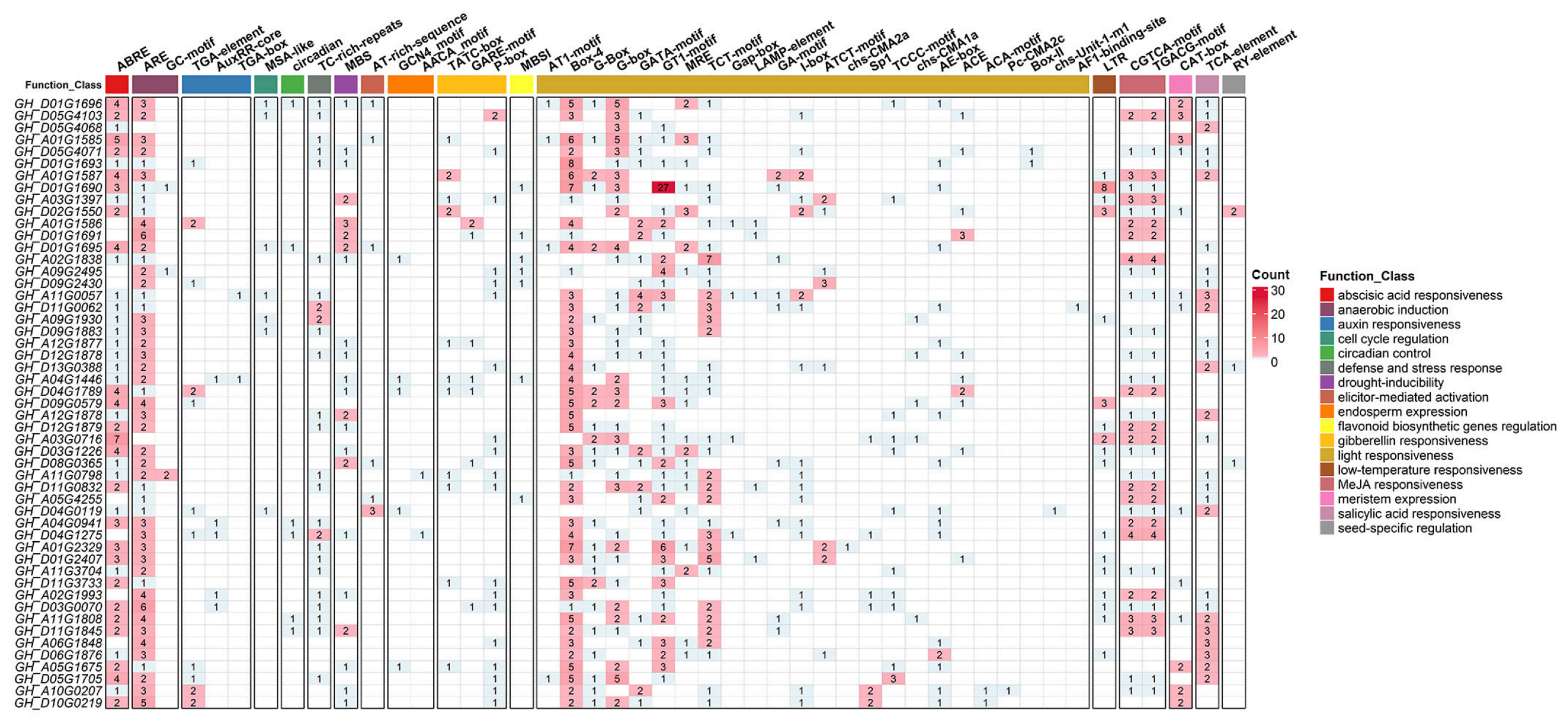
Analysis of cis-acting elements within promoter regions of *GhFWLs*. (Distinct cis-acting elements are represented by different colors. The number inside each cell indicates the count of corresponding cis-elements harbored by each gene. Full names for the abbreviated functional classes shown in the top panel are listed in the right panel).

To explore the transcriptional regulation mechanism of the *GhFWL* gene in *G. hirsutum*, the cis-acting elements within their promoter regions were predicted and analyzed (Figure 4). A total of 47 distinct cis-acting elements were identified and classified into 11 functional classes. Among them, 24 cis-acting elements were involved in light responsiveness, and each *GhFWL* promoter possessed at least two different light-responsive elements, suggesting *GhFWLs* may take part in multiple light-regulated pathways. Elements associated with developmental regulation, including cell cycle regulation, endosperm expression, meristem expression, and seed-specific expression, were detected in 7, 7, 13, and 3 *GhFWL* promoters, respectively. Additionally, hormone-responsive elements corresponding to abscisic acid (ABA), auxin, gibberellin, jasmonic acid, and salicylic acid were identified. Specifically, 84.3% (43) of *GhFWL* promoters contained at least one ABA-responsive element, while 34.1% (16) of *GhFWL* promoters contained auxin-responsive elements. Elements involved in defense and stress response, drought-inducibility, and low-temperature responsiveness were also detected in 25, 22, and 17 *GhFWL* promoters, respectively. Collectively, these results suggested that *FWL* gene family members in cotton may play significant roles in mediating hormone signaling pathways and responding to diverse abiotic stresses.

### 3.5. Expression Patterns of GhFWLs and Their Association with Fiber-Related Traits

**Figure 5:**
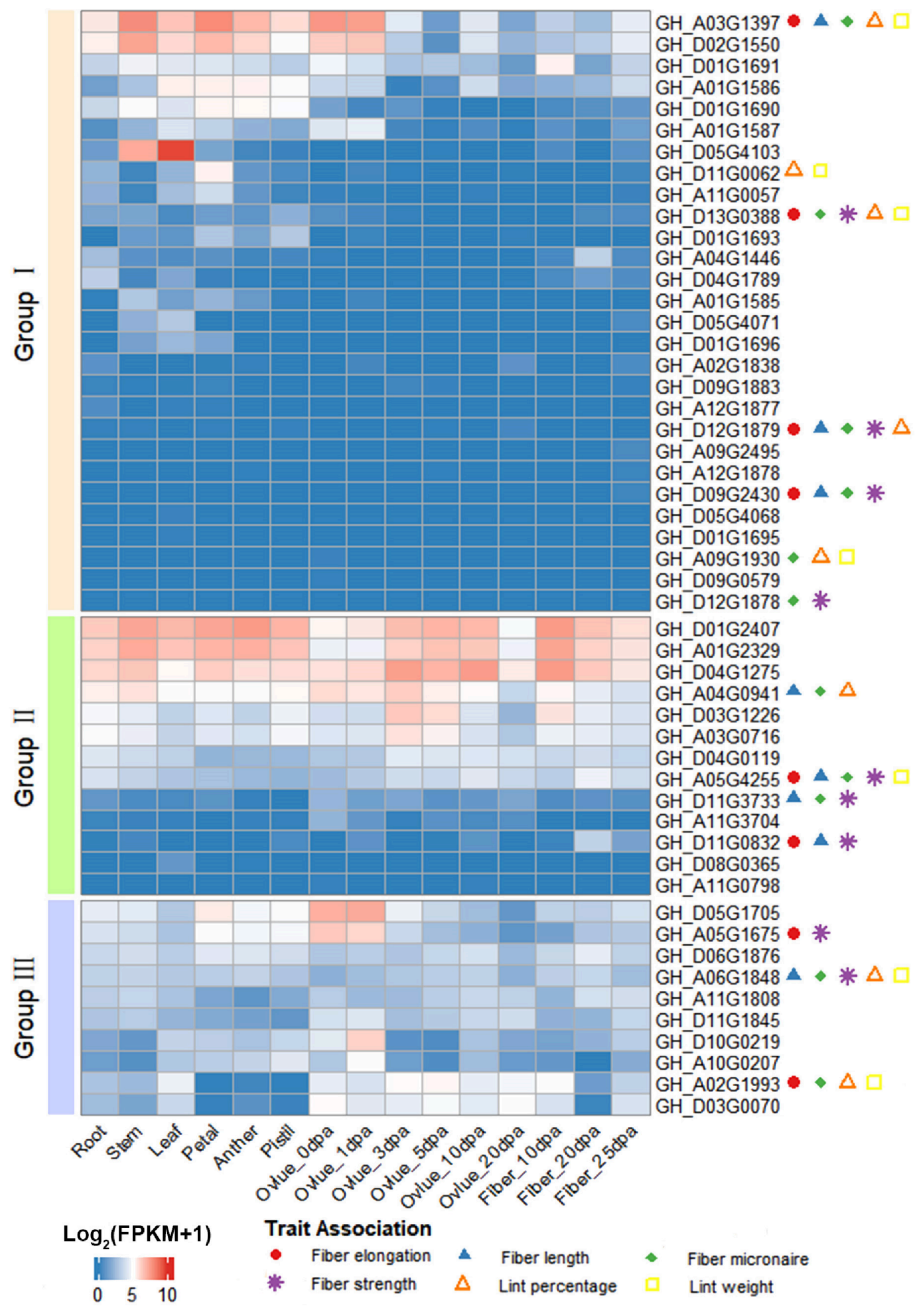
Expression patterns of *GhFWLs* across tissues and their association with fiber-related traits. (*GhFWL* genes were sorted based on their phylogenetic relationships. Relative expression levels across tissues and genes were visualized in the heatmap. Symbols of distinct shapes alongside each gene ID indicate traits significantly associated with the corresponding gene).

To gain insights into the biological functions of *GhFWLs*, their expression patterns were analyzed based on publicly available RNA-seq data from the *G. hirsutum* genetic standard line TM-1. Tissues including the root, stem, leaf, petal, anther, and pistil, as well as developing ovules and fibers, were analyzed (Figure 5). Overall, the expression profiles of *GhFWLs* exhibited diversity and group-specific characteristics. Members from Group III were expressed broadly across almost all tissues, with two of them (*GH_D05G1715* and *GH_A05G1675*) predominantly expressed in ovules at 0 and 1 days post-anthesis (DPA), suggesting their potential roles in regulating fiber initiation. In Group II, expression levels were generally comparable across different tissues within each gene but varied among group members. Group II members could be further clustered into three categories based on their high, moderate, and low expression abundance. Nearly half of Group I members displayed extremely low or undetectable expression levels across all tissues. Additionally, a considerable percentage of group members were preferentially expressed in tissues other than ovules or fibers. These findings suggested that the biological functions of *GhFWLs* might have undergone functional diversification in *G. hirsutum*.

The association between *GhFWLs* and six fiber-related traits was further analyzed to identify their potential roles in fiber development. In total, 14 *GhFWLs* were detected to be significantly associated with fiber-related traits (Figure 5, Table S6). Among them, five *GhFWLs* (*GH_D12G1879*, *GH_D09G2430*, *GH_A05G4255*, *GH_D11G3733*, and *GH_A06G1848*) were simultaneously associated with fiber length, micronaire, and strength, and another six *GhFWLs* (*GH_A03G1397*, *GH_D11G0062*, *GH_D13G0388*, *GH_A09G1930*, *GH_A06G1848*, and *GH_A02G1993*) were co-associated with the two fiber-yield traits, lint percentage and lint weight per boll. Collectively, these results suggested that *FWL* gene family members are candidate regulators in regulating the development of cotton fiber, a specialized tissue in the plant kingdom.

### 3.6. Expression Profiles of GhFWLs Under Abiotic Stress

**Figure 6:**
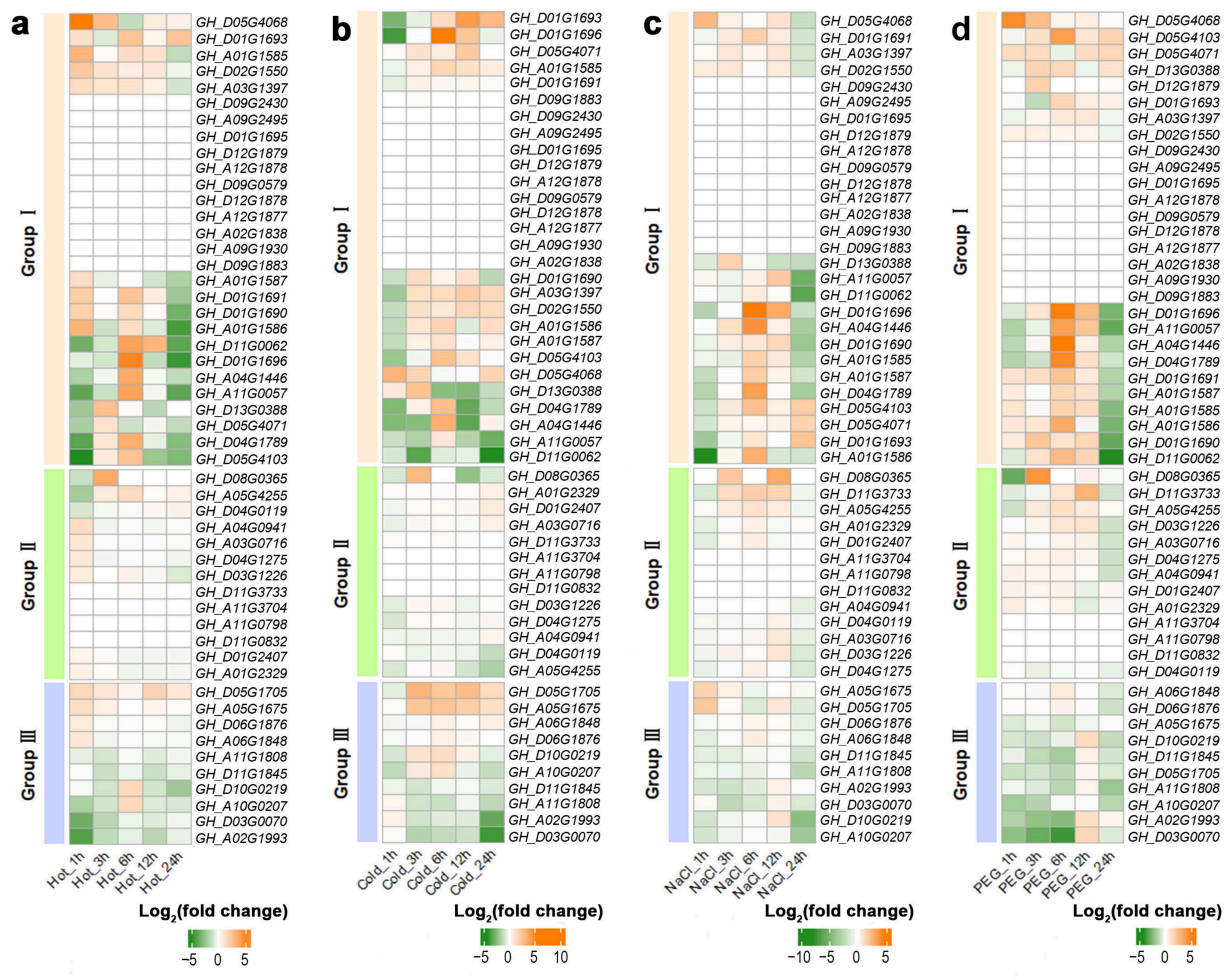
Dynamic expression changes in *GhFWL* genes under abiotic stress. (**a**–**d**) Dynamic expression changes in *GhFWL* genes under heat (**a**), cold (**b**), salt (**c**), and drought stress (**d**), respectively. Heatmaps were generated based on log_2_(fold change) values.

To investigate the potential roles of *GhFWLs* in abiotic stress responses, their expression profiles were analyzed using the publicly available RNA-seq data under stress treatments, including heat, cold, salt, and drought. The results indicated that many *GhFWLs* responded to these stresses. In general, *GhFWLs* in Group II were the most weakly induced under all four conditions, followed by those in Group III, whereas the Group I members showed the strongest induction. Under heat stress, the majority of *GhFWLs* in Group III were down-regulated throughout the treatment period, while a few *GhFWLs* in Group I exhibited sustained up-regulation (Figure 6a). In response to cold stress, the expression of most *GhFWLs* declined rapidly at the early stage, followed by an increase in some of them at later time points. This phenomenon was observed in both Group I and Group III (Figure 6b). Upon salt and drought stress treatment, the expression of *GhFWLs* in Group III tended to remain down-regulated, while *GhFWLs* in Group I were significantly up-regulated during the treatment period (Figure 6c,d). Collectively, these findings suggested that cotton *FWL* gene family members play important and diverse roles in abiotic stress responses, with different groups exhibiting distinct expression patterns under various stress conditions.

### 3.7. Functional Validation of GH_A02G1993 in Response to Salt Stress

**Figure 7:**
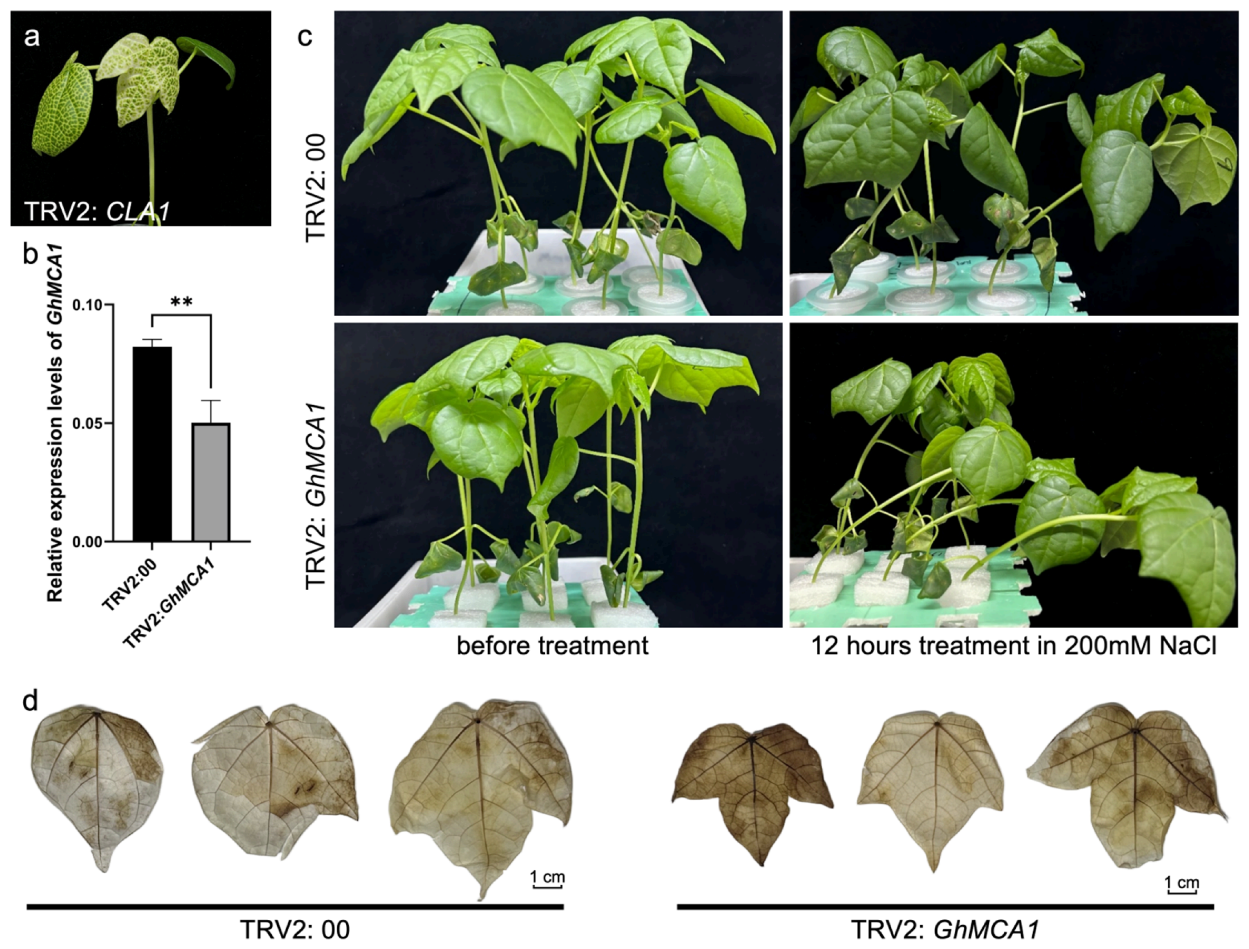
Functional validation of *GhMCA1* in response to salt stress. (**a**) Silencing efficiency indicated by the albino phenotype in TRV2:*CLA1* plants. (**b**) Relative expression levels of *GhMCA1* in TRV2:00 and TRV2:*GhMCA1* seedlings. ** indicate significant differences at *p* < 0.01. (**c**) Phenotype of TRV2:00 and TRV2:*GhMCA1* seedlings before and after salt stress for 12 h; the left panel shows untreated controls. (**d**) H_2_O_2_ accumulation in seedling leaves detected via DAB staining after 12 h of salt treatment.

To further investigate the role of the *FWL* gene family in the abiotic stress response of upland cotton, we selected a specific family member in Group III, *GH_A02G1993* (hereinafter referred to as *GhMCA1*, named after its *Arabidopsis* homolog), for functional validation via the VIGS assay. This gene contains an extra MCA domain alongside the conserved PLAC8 domain and was down-regulated in response to salt stress. The high efficiency of VIGS was confirmed by the albino phenotype in the positive control plants (Figure 7a) and a significant reduction in *GhMCA1* transcript levels in the TRV2:*GhMCA1* lines compared to the TRV2:00 negative control (Figure 7b). After 12 h of salt treatment, the TRV2:*GhMCA1*-silenced plants exhibited severe wilting symptoms, while the negative control plants (TRV2:00) suffered milder salt-induced damage (Figure 7c). In parallel, DAB (3,3′-Diaminobenzidine) histochemical staining showed much darker brown staining of the silenced plant leaves than control plants under salt stress (Figure 7d), reflecting massive accumulation of H_2_O_2_ and aggravated oxidative injury in *GhMCA1*-silenced lines under salt treatment. These results demonstrate that *GhMCA1* participates in the salt stress response in cotton plants. The compromised salt tolerance observed following *GhMCA1* silencing further implied that this gene functions as a positive regulator of salt tolerance.

## 4. Discussion

Members of the *FWL* gene family, which are highly conserved across eukaryotes, have been widely demonstrated to play significant roles in regulating cell division [10,12,42,43,44] and cell size [11], as well as in regulating plant response to diverse stresses [15,19,20,22,45]. *FWL* gene family members have been identified on a genome-wide scale in various plants, including the monocots rice [21], maize [10], and sorghum [10], as well as the dicots tomato [22] and soybean [46]. However, hardly any research on the *FWL* gene family has been conducted in the *Gossypium* species. Moreover, the potential role of *FWL* genes remains largely unexplored in controlling the development of cotton fiber, which is a species-specific tissue.

In the present study, 51 *FWL* gene members were identified in *G. hirsutum*, the most widely cultivated cotton species. However, the number of *GhFWL* genes localized in the At subgenome (22) and the Dt subgenome (29) differs greatly, despite the larger subgenome size of the At subgenome compared to the Dt subgenome [26]. This result differed from the majority of gene families such as the isopentenyl transferase (IPT) family [23], the heat shock protein 90 (HSP90) family [24], and the Early Flowering 4 family [25], which showed comparable member numbers between the subgenomes of upland cotton. One reason for the greater number of *GhFWLs* in the Dt subgenome was the tandem duplication that occurred on chromosome D05 but not on its homeologous chromosome A05. Additionally, the gene loss events that took place in the At subgenome following the polyploidization of *G. hirsutum* were also a reason for this numerical disparity. This finding was consistent with the previously demonstrated evidence that the At and Dt subgenomes of tetraploid cotton have undergone asymmetric domestication [47].

In addition to the tandem duplication on chromosome D05, two further tandem duplication events occurred on chromosomes A01 and D01. Notably, *GH_A01G1586* and *GH_D01G1691*, which are localized within these tandem arrays, each possess an extra PLAC8 domain in comparison to other members, suggesting that they themselves are products of a duplication event. The presence of similar tandem duplications on the homologous chromosomes A01 and D01 suggested that these duplication events occurred before the polyploidization of cotton species. This provided evidence for a specific expansion of the *FWL* gene family in cotton, which may have contributed to environmental adaptation or the regulation of important agronomic traits. Furthermore, segmental duplication was the primary reason for family expansion in cotton, as 82.4% (42) of *GhFWLs* identified through collinearity analysis possess syntenic orthologs in at least one other species. This result was consistent with the report for tomato [22].

Compared with previous reports, the *FWL* family in upland cotton exhibits greater expansion than in *Arabidopsis*, soybean, and tomato, which is consistent with polyploidy and duplications. Additionally, Ka/Ks ratio analysis offered further insights into the evolutionary constraints imposed on the *FWL* family in cotton species. The majority of homologous gene pairs display a Ka/Ks below 0.5, suggesting that purifying selection serves as the primary evolutionary force driving the evolution of this gene family. This observation is consistent with the fact that the PLAC8 domain exhibits high conservation among eukaryotes [4,14,22], while the three pairs with a Ka/Ks ratio larger than 1 might be candidates for further investigation into specialized functions.

Responsiveness to various abiotic stress conditions is a conserved characteristic of the *FWL* gene family [19,22,45,48]. Consistent with previous reports, our study also showed that cis-acting elements related to stress response widely exist within the promoters of *GhFWLs*. Expression analysis further indicated that *GhFWLs* are widely induced by abiotic stresses. However, members from different phylogenetic groups exhibited distinct expression patterns in response to stress. In addition to revealing functional diversity among its phylogenetic groups, these findings confirmed the functional conservation of the *FWL* gene family in cotton. Additionally, by combining the analysis of expression patterns and cis-acting elements, we speculated that the family members respond to abiotic stress through different mechanisms. Several members might be directly induced via their stress-responsive cis-acting elements. For instance, *GH_D01G1690* and *GH_D02G1550*, as well as their homologous pairs, each possess at least one low-temperature responsiveness element and display nearly identical expression patterns under cold stress. However, some *GhFWLs* that possess no stress-responsive elements, such as *GH_D05G4068* and *GH_D13G0388*, are still induced. This induction might be mediated by plant hormones that relay signals through hormone responsiveness elements in their promoters. Additionally, many family members contain both stress- and hormone-responsive cis-elements, implying that their induction may be driven by the concerted action of both mechanisms. However, the analysis of these cis-elements was based on predictions, and further direct experiments are still required to confirm their biological functions.

Among *GhFWL* members of the three groups, those in Group III exhibited distinct characteristics, including additional motifs and domains, as well as unique gene structures. This finding suggested that the biological functions of GhFWL proteins from Group III may differ from those in Group I and Group II. *AtMCA1* and *AtMCA2* from *Arabidopsis*, which have been reported as unique members of the *FWL* gene family with specialized functions [49,50,51], were also clustered in Group III. Apart from the PLAC8 domain, MCA proteins possess an MCAfuc domain, which is structurally similar to the EF-hand domain and is correlated with Ca^2+^ influx [4,42]. Previous studies have shown that the MCA proteins have taken part in the regulation of both stress response [16,17] and organ size control [11,44]. In this study, four GhFWL proteins with the MCAfuc domain were identified, and they were strongly responsive to abiotic stresses based on expression analysis. Our functional validation of *GhMCA1* using the VIGS assay provided further evidence for the role of this gene in salt stress response. Combined with the prediction that the GhMCA1 protein is localized to the plasma membrane (Table S1), we speculated that the underlying mechanism of this response might also be associated with its role in Ca^2+^ signaling, although direct experimental evidence for this link is currently lacking. However, VIGS provides rapid functional insight, but it has inherent limitations. Stable transformation experiments are therefore required to validate this hypothesis and elucidate the precise mechanisms in cotton. Furthermore, our association test showed that *GhMCA1* was significantly associated with fiber elongation, fiber micronaire, lint percentage, and lint weight per boll. Since each fiber is a single cell that differentiates from the seed coat epidermis, fiber yield and quality are strongly influenced by processes governing cell development. MCA1 proteins have been reported to regulate cell proliferation through hormone-mediated pathways in maize [11] and via Ca^2+^ signaling in *Marchantia polymorpha* [44]. The observed associations raise the hypothesis that *GhMCA1* may influence fiber development, potentially through mechanisms analogous to those reported for MCA homologs in other species. However, this hypothesis requires direct experimental testing.

Many *FWL* genes have been demonstrated to regulate organ size, which is similar to *fw2.2*, the first family member identified in tomato [9,10,12,19,52]. Although this highly conserved function has been verified in various plants, the underlying molecular mechanism remains largely unknown. A recent study on *fw2.2* revealed its role in regulating callose deposition at plasmodesmata [8]. During the elongation of cotton fiber cells, callose accounts for 10% of the cell wall mass, and it acts as a precursor of cellulose, which accounts for more than 90% of the secondary cell walls of mature cotton fiber [53,54]. Our association analysis identified 14 *GhFWLs* that exhibited significant associations with fiber-related traits. The regulation of fiber development by these genes might also be related to callose deposition and cellulose synthesis, although this hypothesis requires further verification.

Systematic and comprehensive characterization of the *FWL* gene family in this study provided valuable insight into understanding the biological functions of this family in cotton, but it also had limitations. On the one hand, the expression profiles presented in this study were derived entirely from publicly available RNA-seq datasets without independent RT-qPCR validation. Additional quantitative validation would further strengthen the conclusions drawn from the expression analyses. On the other hand, the association analysis between *GhFWL* haplotypes and fiber-related traits provided only initial evidence suggesting that some *GhFWLs* might be candidate regulators in fiber development, but direct causal evidence will still be necessary to further confirm their functions. Furthermore, our VIGS-based functional validation targeted only one of the family members, and the remaining members remain to be characterized. Despite these limitations, our study provides a valuable foundation for future research. *GhFWL* candidates with potential roles in regulating fiber development and abiotic stress responses could be validated primarily via stable transformation methods and eventually applied in the molecular design breeding of elite cotton germplasm.

## 5. Conclusions

In summary, this study provided a comprehensive characterization of the *FWL* gene family in cotton, representing the first systematic analysis of this family in *G. hirsutum*. We identified 51 *GhFWL* genes that underwent tandem and segmental duplication during evolution and classified them into three phylogenetic groups with distinct structural features and expression profiles. Association analyses across two natural populations identified several *GhFWLs* as candidate regulators of fiber-related traits, while transcriptome profiling revealed that many members are responsive to abiotic stress. Functional validation through VIGS confirmed that *GhMCA1* plays a role in mediating salt stress response, supporting the validity of our bioinformatics-based predictions. Collectively, this study enriches the understanding of the *FWL* gene family in cotton and provides candidate genes and foundational information that inform future genetic improvement of fiber yield and stress tolerance.

## Data Availability

All datasets generated and analyzed in this study are provided within the article and its Supplementary files. Further inquiries could be directed to the corresponding author.

## Supplementary Materials

The following supporting information can be downloaded at: https://www.mdpi.com/article/10.3390/biology15151282/s1, Table S1: The physicochemical properties of FWL proteins in *G. hirsutum*; Table S2: The physicochemical properties of FWL proteins in *G. herbaceum*; Table S3: The physicochemical properties of FWL proteins in *G. raimondii*; Table S4: The Ka/Ks value of FWL homologous pairs in cotton species; Table S5: Sequence of primers used in this study; Table S6: Detail information on association results between *GhFWL*s genes and fiber-related traits. Figure S1: Chromosome distribution of *FWL* family members in *G*. *herbaceum* (left) and *G. raimondii* (right). Chromosomes were ordered by their respective chromosome numbers within each species. Gene density across chromosomes was represented by a color gradient. Orange dots marked the physical positions of *FWL* genes. Figure S2: Detailed information on motifs. Motifs 1–10 are arranged from top to bottom in this figure.
